# I and My Friends are Good People: The Perception of Incivility by Self, Friends and Strangers

**DOI:** 10.5964/ejop.v12i1.937

**Published:** 2016-02-29

**Authors:** Ursula Hess, Michel Cossette, Shlomo Hareli

**Affiliations:** a Humboldt-University, Berlin, Germany; b Department of Human Resources Management, HEC, Montreal, Canada; c University of Haifa, Haifa, Israel; University College Cork, Cork, Ireland

**Keywords:** perceived incivility, self-serving-biases, restitution behaviors, forgiveness, emotions

## Abstract

Three studies were conducted to assess self-serving biases in participants’ beliefs about incivility, its antecedents and consequences as well as restitution behaviors and forgiveness as a function of whether a behavior was performed by themselves, strangers or friends. Participants who imagined themselves in the active role not only described their own behavior as more excusable, congruent with an actor-observer bias, but more importantly, they showed strong self-serving biases with regard to all their reactions to the situation – even though this leads to logical contradictions. This self-serving expectation generalized to friends and contrasted sharply with expectations for strangers, whose behaviors were described as logically consistent. The difference between what is expected from self and friends and what is expected from others may account for much of the popular moral outrage at incivility in various social realms.

There is a popular perception that people in general show rudeness across a variety of relationships and that they tend to do so more than oneself (e.g., ABC News “20/20”, 2006). The consequences of varied uncivil behaviours, such as checking emails or texting during meetings, not listening, withholding information or avoiding someone, spreading rumors about colleagues in the office and untidiness in a shared kitchen (Johnson & Indvik, 2001; Porath & Pearson, 2010), have been mainly studied in the work context, where they are seen as one important source of interpersonal conflicts (Pearson, Andersson, & Porath, 2000). Here the notion of norm violation has been emphasized, as incivility in the workplace entails the violation of workplace norms for mutual respect (Andersson & Pearson, 1999).

However, as noted in the ABC poll referred to above, behaviors that violate norms for respect are not uncommon outside the workplace. Yet, there is a strange lack of research on uncivil behaviors outside the workplace. In fact, the closest would be research on a related issue – verbal impoliteness – in the framework of politeness theory (Brown & Stephen, 1987). This research focuses on the specific verbal strategies that can be used to convey a message in more or less polite ways. Uncivil *behavior*, by contrast, has rarely been studied in this framework (e.g., Ambady, Koo, Lee, & Rosenthal, 1996) or only as a means to “qualify” the verbal message (Trees & Manusov, 1998).

A line of research that deals with norm violations is research on moral evaluations and the emotions that are elicited in response to these. In this context, three domains can be distinguished, purity, justice, and community roles linked to emotional reactions of disgust, anger and contempt, respectively (Rozin, Lowery, Imada, & Haidt, 1999). In the form of empathic anger, anger can also be a reaction to the perceived harm that an immoral act may cause (Batson et al., 2007). However, the acts that are typically referred to by incivility – at least when they do not cumulate – are not of the kind to violate norms of purity or justice or cause harm. Rather, they violate social scripts, the general expectations that people have about the proper behavior of others. Even though social norm expectations are often perceived as “right” and “good” (Hull, 1952) their violation is unlikely to result in harm.

Another line of research that has addressed issues related to incivility in the widest sense, is research on the self-serving biases, that is, the tendency to describe oneself as more moral, cooperative, considerate as well as less impolite and unethical than others (Alicke, 1985; Allison, Messick, & Goethals, 1989; Dunning, Meyerowitz, & Holzberg, 1989; Goethals, Messick, & Allison, 1991).Thus, when asked to rate how polite or impolite they are relative to the average person, (e.g., Alicke, 1985), participants describe themselves in a more positive light than the average person. However, in this line of research people do not so much describe what in fact they would do in a specific situation, but rather how they would behave compared to a general standard with regard to an abstract criterion.

The present research aimed to study the perception of incivility outside the workplace by comparing naïve theories about own behavior with naïve theories about the behavior of strangers and friends. That is, we were interested in the social role norms and naïve theories that underlie reactions to incivility.

Two questions were assessed. First, how do descriptions of own reactions to incivility differ from descriptions of others’ reactions to the same incivility? And second, how do people perceive their own uncivil behavior in comparison to the uncivil behavior of others? Importantly, we are less interested in the evaluation of the uncivil behavior as such, than in the normative consequences that are drawn from the initial behavior. Specifically, an uncivil gesture does not stand alone. Initially, the recipients of uncivil behavior usually feel hurt (Johnson & Indvik, 2001). This may lead to an apology by the perpetrator as apologies are effective in repairing relationships and in the reduction of negative feelings towards the offending other (Hareli & Eisikovits, 2006). In turn, the victim may or may not accept such an apology. However, even though there is research on apologies and forgiveness (Gonzales, Pederson, Manning, & Wetter, 1990; Itoi, Ohbuchi, & Fukuno, 1996; Takaku, Weiner, & Ohbuchi, 2001), the whole chain of events has not been studied. Yet, the different steps of the chain of events can all be perceived differently depending on who is the perpetrator of the incivility.

As regards the perception of the uncivil act, we expect, based on research on self-serving biases, that people may construe their own behavior as less uncivil than the same behavior enacted by others, but even if they do not, they may still view themselves as less responsible for it. Specifically, the undesirable behavior would be construed as more situationally driven, more temporally unstable, as well as less controllable (Bradley, 1978; Weiner, 1986) and hence participants would see themselves as less blameworthy and more driven by the particularities of the specific situation.

Moreover, a closely related bias, the actor-observer bias, suggests that although observers tend to abstain from taking responsibility for their own undesirable acts, they do see others as responsible for similar acts (Jones & Nisbett, 1972; Ross, 1977). Based on this rationale, we expect that own behavior, albeit acknowledged as uncivil, would be construed as accidental or uncontrolled and therefore as less threatening to the self (Alicke, 1985).

As regards the chain of events following an incivility, it is interesting to speculate on how these behaviors are connected in people’s mind. The logical chain of events presumes that if a person judges another’s behavior as more uncivil than their own, will they also consider themselves to suffer more and to be more reluctant to forgive this more grievous offense. However, alternatively, the self-serving bias may operate such that people are inclined to claim more favorable reactions for themselves with regard to every individual aspect of this chain of events. This would then result in a logical break where the more grievous misbehavior by a stranger results in less hurt and a more ready acceptance of an apology as well as forgiveness, than would be expected from a stranger in response to one’s own much less grievous act.

## Overview and Hypotheses

The present research had the goal to investigate the norms and naïve theories that guide perceptions of incivility. We expected that the initial act would be rated as more uncivil when the perpetrator is a stranger. We further investigated expected reactions to the act in terms of experienced hurt and forgiveness. We predicted that participants would show a general self-serving bias for all of the individual components of the chain of events such that people’s naïve theories about such an event become logically disconnected.

Specifically, if I feel that someone else’s uncivil act is worse than mine, then I should feel more hurt, and less willing to accept an apology. However, the logic of self-serving biases outlined above suggests the possibility that this logical chain will be disrupted such that at the same time that I feel another’s transgression to be worse, I would also feel less hurt and more forgiving – because each of these separately suggests that I am somehow “better.”

A further goal of the present research was to assess whether such a bias generalizes to friends. That is, is an incivility committed by a friend perceived like one committed by self or rather like one committed by a stranger? Again, there are two possible outcomes.

On one hand, a friend should be perceived as similar to oneself as shared interests, attitudes and behaviors are the ingredients of friendship (Sullivan, 1953) and people are aware of this fact (Davis & Todd, 1985). Thus, people might find it harder to admit wrong-doing in the case of friends as it would reflect negatively on them as well. On the other hand, relationships with friends also imply high levels of caring and respect (Newcomb & Bagwell, 1995). Thus, because of the general expectation of respect among friends, people may feel more hurt by uncivil friends, but, because of the friendship be nonetheless more inclined to forgive them. This may suggest that judgments would be even more self-biased when the perpetrator of a rude act is a friend.

To address these questions we used a vignette approach. The use of vignettes has been criticized because they represent a reality that is different from the more stimulus rich and interactive environment of actual social interactions (see for example, Parkinson & Manstead, 1993, for a discussion of this issue). On the other hand, vignettes are an excellent tool to assess symbolic knowledge about the social norms that people apply when judging social interactions (Hareli, Shomrat, & Biger, 2005; Robinson & Clore, 2002). As the present study is principally concerned with assessing the norms and naïve theories that are relevant to the perception of incivility outside the workplace, the use of vignettes was considered appropriate.

Three vignette studies were conducted. In Study 1, the imagined interaction partner was a stranger, whereas in Study 2 the imagined interaction partner was a friend. Study 3 assessed reactions to the same act when the imagined interaction partner was either a friend or a stranger. In all three studies we assessed perceptions of incivility from the perspective of the ‘victim’ versus the ‘perpetrator’. The extent to which the behavior was attributed to external, uncontrollable, and temporary causes was assessed in each case. To investigate the reactions to the initial behavior we explored perceptions of hurt caused by the behavior and how likely ‘victims’ and ‘perpetrators’ considered restitution behaviors on the part of the perpetrator as well as their acceptance by the victim. Importantly, each participant responded only to one of the scenarios and hence no explicit comparison with the abstract “average person” was invited.

## Study 1

### Method

#### Participants

A total of 212 participants (71 men) with a mean age of 29 years (*SD* = 10) were recruited using a snowball system. For this, research assistants sent out invitations to participate in a web based research project and asked the participants to then send the invitation on to other people.

#### Vignettes

Two vignettes were selected from a list of situations that had been reported by research assistants as recent incivilities that they had encountered and for which there was agreement that they represent typical uncivil behavior by strangers. Vignette 1 described a protagonist who cuts off another car at a highway exit and Vignette 2 described a person who speaks increasingly loudly into their cell phone while sitting in a bus opposite another person. The vignettes were written from either a 3rd person (i.e., the *driver of the other car* cut me off at the exit) or a 1st person perspective (i.e., *I* cut another driver off at the exit). The transcripts of the vignettes for this as well as for the other studies appear in the Appendix.

#### Dependent Measures

##### Perceived incivility

Three items assessed the incivility of the behavior. Participants were asked to rate the behavior on bipolar 11-point scales anchored with illegitimate – legitimate, impolite – polite, and disrespectful – respectful. The three items were combined into a single scale (Study 1: α = .80; Study 2: α = .85, Study 3: α = .76).

##### Dimensions of causal attribution

Participants were asked to indicate on four 11-point bipolar scales to what degree the cause for the event was "located within the self" – "located within the environment’, "permanent" – "temporary", "controllable"– "uncontrollable" (by the protagonist), and "controllable"– "uncontrollable" (by another person) to assess locus, stability, and controllability respectively.

##### Hurt

We assessed the participant’s view of the hurt that the behavior caused the other individual involved. For this, participants rated the victim’s likely emotional reactions in terms of being irritated/frustrated, angry, sad, hurt, disgusted as well as indifferent on 7-point scales anchored with 0 - not at all and 6 – very intensely. The five negative emotion items correlated highly and were combined into a single negative emotion scale (Study 1: α = .70; Study 2: α = .85, Study 3: α = .77).^i^

##### Restitution behaviors and their impact

For this, participants were asked to indicate on 11-point scales anchored with 1 – unlikely and 11 – very likely, the likelihood that the protagonist would apologize and the likelihood that the victim would forgive the protagonist. In addition they were asked about the likelihood that even after receiving an apology the victim would still feel angry.

#### Procedure

Participants were asked to read the vignettes and to respond to the questions. Each participant received only one vignette. The questions were phrased to reflect the role of the participant as either victim or perpetrator and presented in the order above.

### Results

Participants who imagined themselves as perpetrators, (*M* = 4.52, *SD* = 2.11), compared to those who imagined themselves as victims (*M* = 3.74, *SD* = 1.92), *t*(210) = 2.81, *p* < .01, *d* = .39, rated their behavior more positively on the incivility scale. This perception is congruent with the causal attributions that participants made as a function of their specific role. That is, participants in the role of the perpetrator of an incivility considered the cause of “their” behavior as more temporary (*M* = 8.71, *SD* = 2.54 versus *M* = 7.03, *SD* = 3.00) and situation driven (*M* = 6.00, *SD* = 3.25 versus *M* = 5.25, *SD* = 3.01) than those who rated a stranger’s incivility toward them, *t*(210) = 4.31, *p* < .001, *d* = .59 and *t*(210) = 1.74, *p* = .084, *d* = .24, respectively.

However, participants did not consider the situation to be less controllable by themselves when they were the protagonist than when they were the victim in the story (*M* = 3.79, *SD* = 2.87 versus *M* = 3.93, *SD* = 2.93), *t*(210) = .35, *p* = .728, *d* = .05; nor more controllable by others (*M* = 7.55, *SD* = 3.05 versus *M* = 7.94, *SD* = 2.91), *t*(210) = .95, *p* = .345, *d* = .13.

To assess the perception of the hurt caused by the behavior we assessed perceptions of the likely negative emotions experienced by the victim. As predicted by the self-serving bias view, participants who imagined themselves in the role of the victim reported feeling less negative emotion (*M* = 1.83, *SD* = .99 versus *M* = 2.38, *SD* = 1.02) *t*(210) = 3.99, *p* < .001, d = .55, but not more indifference (*M* = 2.00, *SD* = 1.72 versus *M* = 1.97, *SD* = 1.56), *t*(210) = .13, *p* = .895, *d* = .02, than did participants who imagined the role of the perpetrator. That is, as victims, participants considered themselves to be less likely to suffer hurt when exposed to inconsiderate behavior than strangers would be in the same situation.

With regard to restitution behaviors, a self-serving bias also emerged. Essentially, participants in the active role (apologizing, accepting an apology, remaining angry) portrayed a more positive view than those in the passive role (receiving an apology, being granted forgiveness, and being the target of residual anger).

Thus, when imagining themselves in the role of the perpetrator of the incivility, participants considered it much more likely that they would apologize (*M* = 6.20, *SD*= 3.56), than participants in the victim role expected strangers to apologize (*M* = 3.39, *SD* = 2.55), *t*(210) = 6.65, *p* < .001, *d* = .91. Conversely, participants in the victim role considered it more likely for themselves to forgive (*M* = 9.47, *SD* = 2.18), than participants in the perpetrator role considered it to be forgiven (*M* = 7.27, *SD* = 3.00), *t*(210) = 6.16, *p* < .001, *d* = .84. Finally, victims believed themselves to be less likely to remain angry (*M* = 3.01, *SD* = 2.33) than perpetrators anticipated victims to be (*M* = 5.06, *SD* = 2.67), *t*(210) = 5.97, *p* < .001, *d* = .82.

### Discussion

As expected, participants who were asked to imagine themselves in the role of the perpetrator of an incivility, considered that act to be less uncivil than participants imagining being at the receiving end of the same act. Congruent with this evaluation, the “perpetrators” considered the behavior to be less permanent and more situational, that is, they described themselves as less responsible for the event, which was conceived of as situationally driven. This reaction is typical for a hedonic bias (Shaver, 1970; Weiner, 1986) such that fault is deflected from the person by attributing the cause to the situation.

More interesting, however, is the divergence between victims and perpetrators when it comes to perceptions of the victim’s emotional reactions to the behavior and to restitution behaviors. Participants' who described aspects of the situation in which actions or reactions were in their own hands perceived the situation more positively. That is, they expected to feel less hurt, to be more likely to apologize and to accept an apology, as well as less likely to remain angry after an apology. Conversely, participants' described aspects of the situation that were in the stranger’s hands considerably less positively. In particular, they considered it much less likely to receive an apology as well as for their own apology to be accepted by the stranger. This pattern suggests that, when compared to unknown others, participants judged themselves as reacting more ideally to the situation than others would, that is, they showed a strong self-serving bias when it comes to reactions to rude behavior. This in turn would imply an implicit norm to “stand above” such things and to readily forgive incivilities.

It is interesting to note that despite the fact that the stranger was seen as more responsible for the incivility and that the behavior was perceived as characteristic of that person, participants described themselves as less hurt, more likely to forgive and less likely to remain angry. Yet, when the “victim” is another person -- who was supposedly hurt accidentally by a situation driven act -- he or she was expected to feel more hurt and to be less likely to forgive and more likely to remain angry. Oneself was also seen as more likely to apologize. That is, participants viewed themselves as better than the other in all respects – thereby disconnecting the logical link between the events that follow a given behavior, which would lead one to expect that purposeful bad behavior would result in more pronounced negative effects than an accidental act.

However, in the present study the incivility was after all committed by a perfect stranger, and one may argue that such acts should indeed not be considered as overly hurtful and be put aside easily as they are unlikely to reoccur. Also, it is easy for a person to see him/her self as better than others whose identity is unknown, as most research on self-serving assessments has considered unknown others (e.g., Alicke, 1985; Epley & Dunning, 2000). But what happens when the incivility is committed by a friend who by definition is more similar to the participants (Davis & Todd, 1985)? This is the question addressed by Study 2.

## Study 2

### Method

#### Participants

A total of 245 participants (75 men) with a mean age of 30 years (*SD* = 11) were recruited using the snowball system described for Study 1.

#### Vignettes

Study 2 employed the same procedure and dependent measures as Study 1. However, the two vignettes described an interaction with a friend. Therefore, different topics were chosen. This was done because it is important for the present study that the behaviors are representative of ones likely to occur in interaction with friends. As it is unlikely to meet a friend on the highway or the bus and that s/he will then behave in the way described in the vignettes used for Study 1, these vignettes are not suitable models for such an interaction. We therefore used the same selection procedure as described for Study 1 to select vignettes for uncivil interactions with friends. Vignette 1 described a situation where in the middle of an important personal discussion with a friend, the protagonist’s cell phone rings and the protagonist then proceeds to talk on the phone for several minutes. Vignette 2 describes a situation where a friend who is invited for dinner backs out at the last minute to accept another invitation. Again, vignettes were written in either the 3rd person perspective (i.e., *my friend* answered the phone) or the 1st person perspective (i.e., *I* answered the phone). Each participant read and responded to only one vignette.

### Results

Contrary to Study 1, participants who imagined themselves as perpetrators of the incivility did not consider the behavior as significantly more positive on the incivility scale (*M* = 3.94, *SD* = 2.36) than participants who imagined themselves as the victim of the incivility by a friend (*M* = 4.38, *SD* = 2.30), *t*(243) = 1.46, *p* = .146, *d* = .19. This is not due to an overall difference in perceived incivility that would have resulted in a floor effect. In fact, the levels of perceived incivility of the behaviors in Study 1 (*M* = 4.01, *SD* = 2.04) and Study 2 (*M* = 4.07, *SD* = 2.34) were nearly identical.

However, participants who imagined the role of the perpetrator nonetheless perceived the causes of the event as more temporary (*M* = 8.72, *SD* = 3.04 versus *M* = 7.54, *SD* = 2.91), *t*(243) = 3.11, *p* < .01, *d* = .40, situation driven (*M* = 6.38, *SD* = 3.52 versus *M* = 5.05, *SD* = 3.11), *t*(243) = 3.15, *p* < .01, *d* = .40, and more uncontrollable by them (*M* = 5.88, *SD* = 3.58 versus *M* = 4.95, *SD* = 3.21), *t*(243) = 2.15, *p* < .05, *d* = .27, but not by another person (*M* = 7.17, *SD* = 3.12 versus *M* = 6.92, *SD* = 3.00), *t*(243) = .65, *p* = .52, *d* = .08. That is, even though perpetrators considered “their” behavior to be as uncivil as did those participants who evaluated a friend’s behavior they still distanced themselves from responsibility for the event.

As regards perceptions of hurt, the same pattern as in Study 1 emerged. That is, participants who imagined themselves in the role of the victim, reported feeling less negative emotion (*M* = 2.59, *SD* = .34 versus *M* = 3.18, *SD* = 1.42), *t*(243) = 3.34, *p* < .01, *d* = .43. In addition, they also expected themselves to be more indifferent (*M* = 1.67, *SD* = 1.51 versus *M* = 1.17, *SD* = 1.39), *t*(243) = 2.71, *p* < .01, *d* = .34, than did participants who imagined the role of the perpetrator. In other words, as victims, participants considered themselves as less likely to suffer when exposed to inconsiderate behavior than their friends would be in the same situation.

However, as regards restitution behaviors, a very different pattern from Study 1 emerged. Whereas in Study 1 participants imagining themselves in the role of the perpetrator considered it much more likely that they would apologize to a stranger, forgive a stranger who apologized and expected to feel less anger after receiving an apology than would be the case vice versa, this pattern was not observed for friends. In fact, friends were described in equally positive terms, in that participants saw themselves and their friends as equally likely to apologize (*M* = 8.67, *SD* = 3.39 versus *M* = 8.52, *SD* = 2.57), *t*(243) = .93, *p* = .355, *d* = .12, to accept an apology (*M* = 9.66, *SD* = 2.14 versus *M* = 9.44, *SD* = 2.02), *t*(243) = .82, *p* = .415, *d* = .11, as well as (un)likely to remain angry (*M* = 3.33, *SD*= 2.72 versus *M* = 3.87, *SD* = 3.03), *t*(243) = 1.47, *p* = 1.44, *d* = .19. Thus, participants extended their positive view of their own likely behavior in such a situation to their friends.

### Discussion

Overall, a very different picture emerged for incivilities involving friends rather than strangers. Incivilities by either oneself or the friend were seen as equally rude and restitution behaviors and reactions were considered as equally likely. Only two differences between self and friends emerged. Participants considered themselves less hurt by the behavior and they attributed less causal responsibility to themselves. Importantly, the lack of differences between self and friends was not due to the level of incivility of the behavior. In fact, the level of perceived incivility (*M* = 4.07) was almost identical to Study 1 (*M* = 4.01).

These findings suggest that the self-serving bias observed in Study 1 for the self largely extends to friends and may be considered to be more of an in-group bias in that context. This observation fits with the notion that friends are in fact perceived as very similar to oneself with regard to beliefs, attitudes and behaviors (Davis & Todd, 1985). Causal attributions in contrast have been found to be insensitive to in-group bias (Smith, Whitehead, & Sussman, 1990), which explains why the actor-observer bias remains unaffected.

However, this conclusion is limited by the fact that friends and strangers were described as engaging in different types of acts. This was done to assure that the situations described would be commonly observed incivilities, which are representative for interactions with friends and strangers respectively. Given that by definition we interact differently with friends and strangers this resulted in separate sets of vignettes. Yet, it would still be interesting to consider how friends and strangers compare when the same act is described even if these behaviors are of necessity less representative and more extreme. This was done in Study 3.

## Study 3

### Method

#### Participants

A total of 435 participants (138 men) with a mean age of 26 years (*SD* = 8) were recruited either using the snowball system described for Study 1 (*N* = 98), or using a paper version distributed in large classes or public places at the University of Quebec at Montreal (*N* = 337).

#### Vignettes

Study 3 employed the same procedure and dependent measures as Study 1. However, for Study 3 vignettes were chosen that described situations that can occur with both friends and strangers as interaction partners. For this, we used the same selection procedure as described for Study 1 to select vignettes for incivilities likely to occur with both friends and strangers. Vignette 1 described a situation where the friend/stranger shares an elevator with the ‘victim’ and sneezes wetly without holding their hand in front of their face. Vignette 2 describes a situation where the friend/stranger walks through a door without holding it open for the closely following ‘victim’ such that the door hits the ‘victim.’ Again, vignettes were written in either the 3rd person perspective (i.e., *my friend*/*a stranger* walked through a door without holding it open for you) or the 1st person perspective (i.e., *I* walked through a door without holding it open for a friend/stranger). Each participant read and responded to only one vignette. As expected these behaviors were rated as considerably less positively on the 11-point incivility scale (*M* = 3.34, *SD* = 1.97), than the situations described in studies 1 (*M* = 4.01, *SD* = 2.04) and 2 (*M* = 4.07, *SD* = 2.34).

### Results

To compare perceptions for victims and perpetrators as a function of the friend or stranger status of the interaction partner, a 2 protagonist (self/other) x 2 relationship (friend/stranger) ANOVA was conducted on all dependent variables. See Table 1 for F-values and Table 2 for means and standard deviations. Simple effects analysis and post-hoc tests were used to follow up on significant effects.

**Table 1:** Analysis of Variance Results for Study 3

Scale		Perpetrator (P)			Relationship (R)			P x R		
	*df*	*F*	*p*	η^2^	*F*	*p*	η^2^	*F*	*p*	η^2^
Incivility	1,429	11.09	.001	.03	17.15	.001	.04	6.48	.011	.02
Temporary	1,419	6.92	.009	.02	14.19	.001	.03	23.48	.001	.05
Situational	1,427	1.39	.239	.00	4.82	.029	.01	4.39	.037	.01
Not controllable by actor	1,412	0.16	.694	.00	9.20	.003	.02	2.23	.136	.01
Not controllable by others	1,427	0.17	.678	.00	0.28	.517	.00	0.42	.517	.00
Hurt felt by the victim	1,431	38.93	.001	.08	25.79	.001	.06	1.11	.293	.00
Indifference felt by the victim	1,429	2.17	.142	.01	4.51	.034	.01	1.23	.267	.00
Likelihood to apologize	1,431	59.24	.001	.12	27.00	.001	.06	32.71	.001	.07
Victim's likelihood to forgive	1,431	39.73	.001	.08	50.57	.001	.11	10.95	.001	.03
Victim's feeling of anger after receiving an apology	1,431	42.24	.001	.09	55.83	.001	.12	1.60	.207	.00

**Table 2:** Means and Standard Deviations as a Function of Protagonist and Relationship (Study 3)

Scale	Perpetrator							
	Other				Self			
	Stranger		Friend		Stranger		Friend	
	*M*	*SD*	*M*	*SD*	*M*	*SD*	*M*	*SD*
Incivility^1^	3.06_a_	1.75	4.28_b_	2.14	2.91_a_	1.79	3.36_b_	1.97
Temporary	6.95_a_	2.91	9.31_b_	2.41	8.70_b_	3.13	8.49_b_	2.95
Situational	5.55_a_	0.33	6.97_b_	0.33	5.86_a_	0.33	5.89_a_	0.33
Not controllable by actor	4.60_a_	3.13	6.12_bc_	3.28	4.97_ad_	3.60	5.49_a_	3.60
Not controllable by others	8.63	2.88	8.66	2.91	8.70	3.04	8.36	3.20
Hurt felt by the victim	2.36_a_	1.40	1.87_b_	1.10	3.23_c_	1.17	2.50_a_	1.33
Indifference felt by the victim	1.75_a_	1.83	2.26_b_	1.73	1.69_a_	1.47	1.85_a_	1.49
Likelihood to apologize	6.27_a_	3.20	9.08_b_	2.56	9.73_b_	2.37	9.59_b_	2.56
Victim's likelihood to forgive	9.80_a_	1.85	10.45_b_	1.27	8.15_c_	2.26	9.94_a_	1.63
Victim's feeling of anger after receiving an apology	3.41_a_	2.91	1.87_b_	1.74	5.33	2.82_c_	3.17_a_	2.68

*Note.* Subscripts are based on LSD < .05. Different subscripts denote a significant difference.

^1^Lower values indicate more incivility.

The protagonist’s behavior was considered to be equally uncivil for all perpetrators except when the perpetrator was a friend in which case the behavior was considered to be less uncivil than own behavior and the behavior of strangers. That is, unlike Study 1, participants did not consider themselves as less uncivil than a stranger. This is likely due to a floor effect as many participants noted that they considered the described behaviors to be very uncivil indeed. Yet, it is also possible that the nature of the uncivil behaviors used in this study is responsible for this effect. It remains for future research to examine this possibility.

As regards attributions of causality, participants considered the behavior of the perpetrator who was a stranger as less temporary, more situation driven and less controllable by the self (but not by others) than the behavior of either self or the friend. As regards perceptions of hurt, the same pattern as in Studies 1 and 2 emerged. Specifically, participants considered themselves as less likely to suffer than either a stranger or a friend, but they also expected their friends to suffer less than a stranger would.

As regards restitution behaviors, the results also match both Study 1 and Study 2. That is, participants consider themselves and their friends as more likely than a stranger to apologize, to forgive, and to not remain angry, with either no difference between them and their friends or an even more forgiving attitude when the perpetrator was a friend. Thus, as in Study 2, participants extended their positive view of the likely behavior in such a situation to their friends and as in Study 1, they consider strangers to be much less likely to ask for or grant forgiveness. In sum, with regard to feelings of hurt and restitution a pattern of self- and friend serving bias emerged where participants who saw themselves as victims of strangers and those who imagined having been uncivil to a friend reported themselves and their friends to be less likely to be hurt, more indifferent, more likely to apologize and to forgive and less likely to remain angry.

## General Discussion

In Studies 1 and 3, a self-serving bias was observed in participants’ assessments of an incivility performed by themselves versus a stranger. Not only, as expected from research in the actor observer bias (Jones & Nisbett, 1972; Ross, 1977), did participants consider themselves to be less uncivil and less responsible for the act, but they also considered their behavior to be more positive in terms of both, being able to put the insult aside and to forgive when being in the role of the victim and of making restitution when in the role of the perpetrator. This finding suggests that people perceive a social norm of not being too upset about the rude behavior of strangers and to readily forgive, as well as an expectation that someone who behaved rudely takes responsibility for the act and tenders an apology.

This normative expectation may be the source of much moral outrage at the inconsiderate behavior of others, as it is accompanied by a uniqueness bias (Goethals, 1987; Mullen, Dovidio, Johnson, & Copper, 1992) in so far as others are perceived as considerably less likely to behave in ways that are congruent with this norm. Thus, with regard to incivilities by oneself and strangers and the reactions to them, participants show a consistent “holier than thou” attitude. Notably, this attitude leads participants to present the somewhat illogical chain of events where the worse offense by another leads to less hurt and more forgiveness on their part, whereas their, accidental and less offensive, behavior leads to more hurt and less forgiveness.

The same is not true for interactions with friends. Even though there is still an actor-observer bias evident in the causal attributions in Study 2 such that participants who imagine the perpetrator role consider themselves as less responsible for their acts than the friend would be in the reverse situation, this bias disappears for the more extreme behaviors in Study 3. With regard to hurt and restitution behaviors the overall pattern suggests that participants in the perpetrator role consider themselves and their friends to suffer less and work harder towards restitution – with incivilities by friends being considered less hurtful and more likely to be resolved, in fact, a very reasonable assumption as this is the basis of what it means to be friends.

This overall pattern of effects does not seem to be due to an ultimate attribution error (Pettigrew, 1979) as attributions were found to differ in a way favoring the self. A more likely explanation for the present finding is that the self-serving bias generalizes to friends and hence becomes more of an in-group bias. That is, in the same vein that people attribute more positive attributes (Allen, 1996; Rustemli, Mertan, & Ciftci, 2000) and behaviors (Beaupré & Hess, 2003), the “proper” reaction to an incivility represents one more positive behavior that is preferentially attributed to in-group members. This implies that if we consider it to be normative for a “good person” to apologize, accept apologies and not hold grudges, then this means that people expect their friends to be good persons as well, but strangers to be quite unlikely to be good persons as the difference in the perceived likelihood of the good behavior between self and strangers is strikingly large. This sort of thinking could be one mechanism to reinforce in-group boundaries. In fact, the ascription of “good behavior” to friends and self and of considerably less “good” behavior to strangers, is reminiscent of Leyen’s infra-humanization theory (e.g., Demoulin et al., 2005; Leyens et al., 2000), which claims that humans are motivated to view out-groups as possessing a lesser degree of humanity than the in-group.

Interestingly, the self-serving biases worked in a way that disrupted the logical connection between the sequence of events following an incivility. Thus, even though participants considered the same behavior more uncivil when performed by a stranger rather than themselves or a friend, they considered themselves to be less hurt by the behavior, and also considered it more likely for themselves and their friends to forgive such a behavior and conversely to apologize for their – less voluntary and more ‘accidental’ own incivility.

Recently research has documented that people’s memory of undesirable actions is also affected by self-serving biases (Bell, Schain, & Echterhoff, 2014). Specifically, the level of immorality of morally questionable behaviors was better remembered when it was associated with personal costs than when it was associated with personal benefits. Although such memory effects cannot explain our results, it is of interest for future research to study if the same may be true of impolite behaviors being differentially remembered as a function of the identity of the protagonist.

The strong difference between what it expected from self and friends and what is expected from strangers, especially with regard to restitution behaviors is striking and may account for much of the popular moral outrage at rude behavior. Specifically, in our modern era more and more of the people we interact with and whom we may perceive as uncivil are in fact strangers and our bias to see strangers as both more uncivil and less likely to apologize may then lead us to see the world as more and more rude (e.g., ABC News “20/20", 2006).

## Appendix

### Vignettes Used in Study 1

1. Vous roulez en voiture sur l’autoroute dans la voie du centre. Vous souhaitez prendre la bretelle de sortie qui n’est pas très loin devant, mais vous dépassez au même moment une autre voiture qui roule dans la voie de droite. Plutôt que ralentir et passer derrière l’autre voiture, vous coupez le chemin de l’autre voiture afin de gagner la bretelle de sortie.
2. Vous roulez en voiture sur l’autoroute dans la voie de droite. Une autre voiture à votre gauche veut gagner la bretelle de sortie qui n’est pas très loin devant. Plutôt que ralentir et passer derrière vous, le conducteur vous coupe le chemin afin de gagner la bretelle de sortie.
3. Vous êtes dans l’autobus. Une personne est assise tout juste en face de vous, la personne à l’air fatigué et cogne des clous. Vous répondez à votre téléphone cellulaire et notez que vous avez de la difficulté à entendre votre interlocuteur et à vous faire entendre. Vous continuez votre conversation et parlez de plus en plus fort afin de vous faire entendre.
4. Vous êtes dans l’autobus. Vous êtes fatigué et vous cognez des clous. La personne d’en face répond au téléphone et a de la difficulté d’entendre et de se faire entendre. La personne continue sa conversation et parle de plus en plus fort afin de se faire entendre.

### Vignettes Used in Study 2

1. Un(e) ami(e) vous invite à souper à son domicile. Tard cette même journée, vous recevez une autre invitation. Vous annulez le dîner sans de vous excuser.
2. Vous invitez un(e) ami(e) à souper à votre domicile. Tard cette même journée, votre ami(e) reçoit une autre invitation. Votre ami(e) annule le dîner sans de s’excuser.
3. Vous discutez avec un(e) ami(e). Alors qu’il vous lui parle d’un sujet très important pour lui, votre téléphone cellulaire sonne. Vous répondez au téléphone et parlez plusieurs minutes avec l’autre personne.
4. Vous discutez avec un(e) ami(e). Alors que vous lui parlez d’un sujet très important pour vous, son téléphone cellulaire sonne. Votre ami(e) répond à son téléphone et parle plusieurs minutes avec l’autre personne.

### Vignettes Used in Study 3

1. Vous marchez en direction de votre lieu de travail et apercevez un(e) de vos amis derrière vous. Vous entrez dans l’immeuble et lâchez la porte au lieu de la retenir. En conséquence, la porte passe très proche de frapper votre ami(e) qui est tout juste derrière.
2. Vous marchez en direction de votre lieu de travail et apercevez un(e) de vos amis qui s’apprête à entrer dans l’immeuble. Vous avez la certitude que votre ami(e) vous ait vu approché. Au moment où vous approchez pour entrer, votre ami(e), qui est tout juste devant vous, lâche la porte au lieu de la retenir. La porte passe très proche de vous frapper.
3. Vous marchez en direction de votre lieu de travail et apercevez une personne derrière vous. Vous entrez dans l’immeuble et lâchez la porte au lieu de la retenir. En conséquence, la porte passe très proche de frapper cette personne qui est tout juste derrière.
4. Vous marchez en direction de votre lieu de travail et apercevez une personne qui s’apprête à entrer dans l’immeuble. Vous avez la certitude que cette personne vous ait vu approché. Au moment où vous approchez pour entrer, la personne, qui est tout juste devant vous, lâche la porte au lieu de la retenir. La porte passe très proche de vous frapper.
5. Vous êtes dans un ascenseur bondé de personnes. Votre ami(e) vous fait face. Soudainement, vous éternuez. Vous omettez de mettre votre main et éclaboussez votre ami(e).
6. Vous êtes dans un ascenseur bondé de personnes. Votre ami(e) vous fait face. Soudainement, votre ami(e) éternue. Il/elle omet de mettre sa main devant lui/elle et vous éclabousse.
7. Vous êtes dans un ascenseur bondé de personnes. Une personne vous fait face. Soudainement, vous éternuez. Vous omettez de mettre votre main devant vous et vous éclaboussez l’autre personne.
8. Vous êtes dans un ascenseur bondé de personnes. Une personne vous fait face. Soudainement, cette personne éternue. Elle omet de mettre sa main devant elle et vous éclabousse.

## References

[r1] ABC News “20/20”. (2006). *Poll: Rudeness in America, 2006: It’s the #@%! cell phones.* Retrieved from http://abcnews.go.com/2020/US/story?id=1574155

[r2] Alicke M. D. (1985). Global self-evaluation as determined by the desirability and controllability of trait adjectives. Journal of Personality and Social Psychology, 49(6), 1621–1630. doi:. 10.1037/0022-3514.49.6.1621

[r3] Allen B. P. (1996). African Americans' and European Americans' mutual attributions: Adjective Generation Technique (AGT) stereotyping. Journal of Applied Social Psychology, 26, 884–912. doi:. 10.1111/j.1559-1816.1996.tb01116.x

[r4] Allison S. T. Messick D. M. Goethals G. R. (1989). On being better but not smarter than others: The Muhammad Ali effect. Social Cognition, 7, 275–295. doi:. 10.1521/soco.1989.7.3.275

[r5] Ambady N. Koo J. Lee F. Rosenthal R. (1996). More than words: Linguistic and nonlinguistic politeness in two cultures. Journal of Personality and Social Psychology, 70(5), 996–1011. doi:. 10.1037/0022-3514.70.5.996

[r6] Andersson L. M. Pearson C. M. (1999). Tit for tat? The spiraling effect of incivility in the workplace. The Academy of Management Review, 24, 452–471.

[r7] Batson C. D. Kennedy C. L. Nord L.-A. Stocks E. L. Fleming D. A. Marzette C. M. Zerger T. (2007). Anger at unfairness: Is it moral outrage? European Journal of Social Psychology, 37(6), 1272–1285. doi:. 10.1002/ejsp.434

[r8] Beaupré M. G. Hess U. (2003). In my mind, *we* all smile: A case of in-group favoritism. Journal of Experimental Social Psychology, 39(4), 371–377. doi:. 10.1016/S0022-1031(03)00012-X

[r9] Bell R. Schain C. Echterhoff G. (2014). How selfish is memory for cheaters? Evidence for moral and egoistic biases. Cognition, 132(3), 437–442. doi:. 10.1016/j.cognition.2014.05.001 24908343

[r10] Bradley G. W. (1978). Self-serving biases in the attribution process: A reexamination of the fact or fiction question. Journal of Personality and Social Psychology, 36, 56–71. 10.1037/0022-3514.36.1.56

[r11] Brown, P., & Stephen, L. (1987). *Politeness: Some universals in linguistic usage*. Cambridge, United Kingdom: Cambridge University Press.

[r12] Davis, K. E., & Todd, M. J. (1985). Assessing friendship: Prototypes, paradigm cases, and relationship description. In S. Duck & D. Perlman (Eds.), *Understanding personal relationships* (pp. 17-38). Thousand Oaks, CA, USA: SAGE.

[r13] Demoulin S. Leyens J.-P. Rodríguez-Torres R. Rodríguez-Pérez A. Paladino P. M. Fiske S. T. (2005). Motivation to support a desired conclusion versus motivation to avoid an undesirable conclusion: The case of infra-humanization. International Journal of Psychology, 40, 416–428. doi:. 10.1080/00207590500184495

[r14] Dunning D. Meyerowitz J. A. Holzberg A. D. (1989). Ambiguity and self-evaluation: The role of idiosyncratic trait definitions in self-serving assessments of ability. Journal of Personality and Social Psychology, 57, 1082–1090. doi:. 10.1037/0022-3514.57.6.1082

[r15] Epley N. Dunning D. (2000). Feeling "holier than thou": Are self-serving assessments produced by errors in self- or social prediction? Journal of Personality and Social Psychology, 79, 861–875. doi:. 10.1037/0022-3514.79.6.861 11138757

[r16] Goethals, G. R. (1987). Fabricating and ignoring social reality: Self-serving estimates of consensus. In J. M. Olson, C. P. Herman, & M. P. Zanna (Eds.), *Relative deprivation and social comparison: The Ontario Symposium* (pp. 135-157). Hillsdale, NJ, USA: Lawrence Erlbaum.

[r17] Goethals, G. R., Messick, D. M., & Allison, S. T. (1991). The uniqueness bias: Studies of constructive social comparison. In J. Suls & T. A. Will (Eds.), *Social comparison: Contemporary theory and research* (pp. 149-176). Hillsdale, NJ, USA: Lawrence Erlbaum.

[r18] Gonzales M. H. Pederson J. H. Manning D. J. Wetter D. W. (1990). Pardon my gaffe: Effects of sex, status, and consequence severity on accounts. Journal of Personality and Social Psychology, 58, 610–621. doi:. 10.1037/0022-3514.58.4.610

[r19] Hareli S. Eisikovits Z. (2006). The role of communicating social emotions accompanying apologies in forgiveness. Motivation and Emotion, 30(3), 189–197. doi:. 10.1007/s11031-006-9025-x

[r20] Hareli S. Shomrat N. Biger N. (2005). The role of emotions in employees' explanations for failure in the workplace. Journal of Managerial Psychology, 20, 663-680). doi:10.1108/02683940510631435

[r21] Hull, C. L. (1952). *A behavior system: An introduction to behavior theory concerning the individual organism*. New Haven, NJ, USA: Yale University Press.

[r22] Itoi R. Ohbuchi K.-I. Fukuno M. (1996). A cross-cultural study of preference of accounts: Relationship closeness, harm severity, and motives of account making. Journal of Applied Social Psychology, 26, 913–934. doi:. 10.1111/j.1559-1816.1996.tb01117.x

[r23] Johnson P. R. Indvik J. (2001). Slings and arrows of rudeness: Incivility in the workplace. Journal of Management Development, 20, 705–713. doi:. 10.1108/EUM0000000005829

[r24] Jones, E. E., & Nisbett, R. E. (1972). The actor and the observer: Divergent perceptions of the causes of behavior. In E. E. Jones, D. E. Kanouse, H. H. Kelley, R. E. Nisbett, S. Valins, & B. Weiner (Eds.), *Attribution: Perceiving the causes of behavior* (pp. 79-94). Morristown, NJ, USA: General Learning.

[r25] Leyens J.-P. Paladino P. M. Rodriguez-Torres R. Vaes J. Demoulin S. Rodriquez-Perez A. Gaunt R. (2000). The emotional side of prejudice: The attribution of secondary emotions to ingroups and outgroups. Personality and Social Psychology Review, 4, 186–197. doi:. 10.1207/S15327957PSPR0402_06

[r26] Mullen B. Dovidio J. F. Johnson C. Copper C. (1992). In-group—Out-group differences in social projection. Journal of Experimental Social Psychology, 28, 422–440. 10.1016/0022-1031(92)90040-Q

[r27] Newcomb A. F. Bagwell C. L. (1995). Children's friendship relations: A meta-analytic review. Psychological Bulletin, 117(2), 306–347. doi:. 10.1037/0033-2909.117.2.306

[r28] Parkinson B. Manstead A. S. R. (1993). Making sense of emotion in stories and social life. Cognition and Emotion, 7, 295–323. doi:. 10.1080/02699939308409191

[r29] Pearson C. M. Andersson L. M. Porath C. L. (2000). Assessing and attacking workplace incivility. Organizational Dynamics, 29(2), 123–137. doi:. 10.1016/S0090-2616(00)00019-X

[r30] Pettigrew T. F. (1979). The ultimate attribution error: Extending Allport's cognitive analysis of prejudice. Personality and Social Psychology Bulletin, 5, 461–476. 10.1177/014616727900500407

[r31] Porath C. L. Pearson C. M. (2010). The cost of bad behavior. Organizational Dynamics, 39, 64–71. doi:. 10.1016/j.orgdyn.2009.10.006

[r32] Robinson M. D. Clore G. L. (2002). Belief and feeling: Evidence for an accessibility model of emotional self-report. Psychological Bulletin, 128, 934–960. 10.1037/0033-2909.128.6.934 12405138

[r33] Ross L. D. (1977). The intuitive psychologist and his shortcomings: Distortions in the attribution process. Advances in Experimental Social Psychology, 10, 173–220. doi:. 10.1016/S0065-2601(08)60357-3

[r34] Rozin P. Lowery L. Imada S. Haidt J. (1999). The CAD triad hypothesis: A mapping between three moral emotions (contempt, anger, disgust) and three moral codes (community, autonomy, divinity). Journal of Personality and Social Psychology, 76, 574–586. doi:. 10.1037/0022-3514.76.4.574 10234846

[r35] Rustemli A. Mertan B. Ciftci O. (2000). In-group favoritism among native and immigrant Turkish Cypriots: Trait evaluations of in-group and out-group targets. The Journal of Social Psychology, 140, 26–34. doi:. 10.1080/00224540009600443 10705667

[r36] Shaver K. G. (1970). Defensive attribution: Effects of severity and relevance on the responsibility assigned for an accident. Journal of Personality and Social Psychology, 14, 101–113. doi:. 10.1037/h0028777

[r37] Smith S. H. Whitehead G. I. III Sussman N. M. (1990). The positivity bias in attributions: Two cross-cultural investigations. Journal of Cross-Cultural Psychology, 21, 283–301. doi:. 10.1177/0022022190213002

[r38] Sullivan, H. S. (1953). *The interpersonal theory of psychiatry*. New York, NY, USA: Norton.

[r39] Takaku S. Weiner B. Ohbuchi K.-I. (2001). A cross-cultural examination of the effects of apology and perspective taking on forgiveness. Journal of Language and Social Psychology, 20, 144–166. doi:. 10.1177/0261927X01020001007

[r40] Trees A. R. Manusov V. (1998). Managing face concerns in criticism: Integrating nonverbal behaviors as a dimension of politeness in female friendship dyads. Human Communication Research, 24, 564–583. doi:. 10.1111/j.1468-2958.1998.tb00431.x

[r41] Weiner, B. (1986). *An attributional theory of motivation and emotion*. New York, NY, USA: Springer.

